# The rationale of using cerebral embolic protection devices during transcatheter aortic valve implantation

**DOI:** 10.1007/s12471-020-01380-7

**Published:** 2020-03-09

**Authors:** C. Simsek, B. E. Schölzel, P. den Heijer, J. Vos, M. Meuwissen, B. van den Branden, A. J. J. IJsselmuiden

**Affiliations:** Department of Cardiology, Amphia Ziekenhuis, Breda, The Netherlands

**Keywords:** Cerebral embolic protection device, Transcatheter aortic valve implantation, Stroke

## Abstract

Aortic valve stenosis is one of the most common valvular abnormalities, which can manifest as angina, syncope, dyspnoea and sudden cardiac death. Transcatheter aortic valve implantation (TAVI) has been introduced as an alternative to surgical valve replacement in patients with severe aortic valve stenosis, resulting in less morbidity, shorter time to recovery and similar mortality rates. Progress in this field has reduced complication rates. However, the incidence of peri-procedural stroke remains relatively high (around 4%). To fully utilise the potential of TAVI, cerebral embolic protection devices (CEPD) have been developed and introduced. In this position paper, we aim to summarise the available data on several CEPD.

## Introduction

**Fig. 1:**
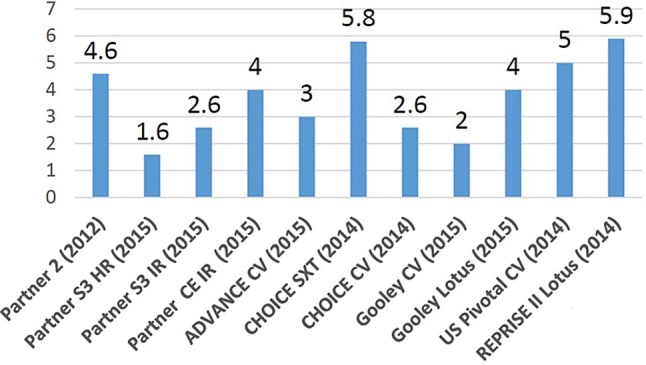
The incidence of Valve Academic Research Consortium (VARC)-2 defined stroke rates in transcatheter aortic valve implantation studies. (Gooley et al. 2015 [11])

Since the introduction of transcatheter aortic valve implantation (TAVI) in 2002, the procedure has become an attractive alternative to surgical valve replacement in the treatment of severe aortic valve stenosis. By 2025, there will be an estimated 280,000 procedures performed worldwide and the total market will exceed 8 billion US$ [1]. Although this highly promising treatment modality results in less morbidity, shorter time to recovery and similar mortality rates, it is still associated with one of the most devastating and feared complications, namely strokes. Newer-generation devices and increased operator experience have reduced the incidence of strokes; however, this still remains relatively high at around 4% (Fig. 1). The majority of these events are procedure-related, taking place immediately post-TAVI and prior to hospital discharge. Additionally, a majority of TAVI patients (between 58 and 100%) have novel silent cerebral lesions as detected by diffusion-weighted magnetic resonance imaging (DW-MRI) [2]. As it may seem that these occult cerebral lesions have no major clinical implication, it is noteworthy that they are associated with a >2-fold increased risk of dementia and faster decline in cognitive function [3]. The implementation of TAVI in a lower-risk population could be limited by the occurrence of these brain injuries. To fully utilise the potential of TAVI, various cerebral embolic protection devices (CEPD) have been developed and investigated in the last few years. These devices provide a mechanical barrier against debris reaching the cerebral circulation by capturing or deflecting emboli to the peripheral circulation. Estimations show that the costs of preventing a single stroke or death will be around 60,000 US$ (21 patients needed to prevent one stroke or death with a CEPD) [4]. Histopathological studies have shown that the composition of the debris varies between thrombus, calcification, tissue from the aortic valve or myocardium, and foreign body components [5]. Approximately a third occurs in the middle cerebral artery distribution, a third in the posterior cerebral artery and a third in the cerebellum/brain stem [3]. Randomised studies did not demonstrate the superiority of CEPD during TAVI procedures for hard clinical endpoints, most likely because these studies were not powered for hard clinical endpoints such as the occurrence of stroke. Although not proven, theoretically the use of CEPD should not only lead to lower stroke rates, but also fewer silent cerebral ischaemic lesions. Determining high-intensity transient signals (HITS) by transcranial Doppler (TCD) measurements during TAVI, which represents solid or gaseous cerebral micro-emboli passing through the middle cerebral artery, could become an important tool in the detection of these emboli, because it has the ability to provide real-time data on blood filtration with a CEPD and provide insights into the critical phases of the TAVI procedure, such as valve positioning, balloon valvuloplasty and deployment of the prosthesis. In this position paper, we aim to summarise the available data on several CEPD.

## Cerebral embolic protection devices

### Sentinel

The Sentinel embolic protection device (Claret Medical, Santa Rosa, CA, USA) is inserted through a 6F sheath from the right upper extremity and consists of two filter baskets (140-µm pores). The proximal filter is positioned in the brachiocephalic trunk and the second filter is inserted into the left common carotid artery. The two filters have a filtering effect of more than 90% of the cerebral blood flow as the territory supplied by the left vertebral artery is unprotected. The operator can achieve full coverage in combination with the Wirion filter (CSI, St. Paul, MN, USA) placed in the left vertebral artery. The Sentinel embolic protection device received FDA approval in 2017 and is to date the most widely used CEPD in TAVI. The device can be deployed in less than 10 min in 90% of cases. The results of the Sentinel trial (*n* = 363 patients) showed that debris was found in 99% of the filters. Despite a reduction in all-cause strokes at 30 days, no statistical significance could be shown (5.6% vs 9.1% in the control group; *p* = 0.25) [6]. The limited power of the study in combination with possible interactions (different valve types, operator experience and pre- or post-dilatations) could all have contributed to this result. The MISTRAL‑C randomised study (*n* = 65 patients) showed fewer new lesions and a smaller total lesion volume (95 mm^3^ vs 197 mm^3^) in the group protected with a CEPD. Also, neurocognitive deterioration was more prominent in the patients treated without the Sentinel device (4% vs 27%, *p* = 0.02) [7]. These results were also found in the CLEAN-TAVI trial (*n* = 100 patients) [8]. In addition, a recently published propensity score-matched analysis by Seeger et al. (*n* = 280 in both groups, after matching) showed that the primary endpoint (composite of mortality or stroke within 7 days) was significantly lower in the CEPD group (2.1% vs 6.8%, *p* = 0.01) [4].

### TriGUARD Embolic Deflection Device

The TriGUARD Embolic Deflection Device (Keystone Heart Ltd., Caesarea, Israel) is designed for use in the aortic arch to reduce the amount of embolic material that may enter the carotid, subclavian or vertebral arteries during endovascular procedures. It consists of a temporary, single-use, biocompatible nitinol filter (130-µm pores), which is delivered transfemorally via a 9F sheath. After being positioned in the aortic arch, it is anchored by a stabiliser in the ostium of the innominate artery. This device was investigated in the DEFLECT I trial, which was a prospective, multicentre, single-arm safety and device functionality study (*n* = 37 patients). Data showed that the presence of new cerebral ischaemic lesions on DW-MRI was similar to historical controls (82% vs 76%, *p* = NS). However, the per-patient total lesion volume was 34% lower compared to historical data (0.2 vs 0.3 cm^3^) [9]. After some modifications had been made to the device, this study paved the way for the randomised DEFLECT III trial (*n* = 85 patients), in which it was found that patients with TriGUARD HDH-protected TAVI procedures had more freedom from ischaemic brain lesions, fewer neurological deficits and improved cognitive functions compared to controls [10]. Although the primary in-hospital procedural safety endpoints (death, stroke, life-threatening or disabling bleeding, stage 2 or 3 acute kidney injury, or major vascular complications) were not statistically different (21.7% of TriGUARD HDH compared to 30.8% control group, *p* = 0.34), it should be noted that this study was explorative and not designed to provide conclusive evidence of the benefit of embolic protection [10]. The results of the REFLECT trial should provide more answers in the near future.

### TriGUARD 3 Embolic Protection Device

**Fig. 2:**
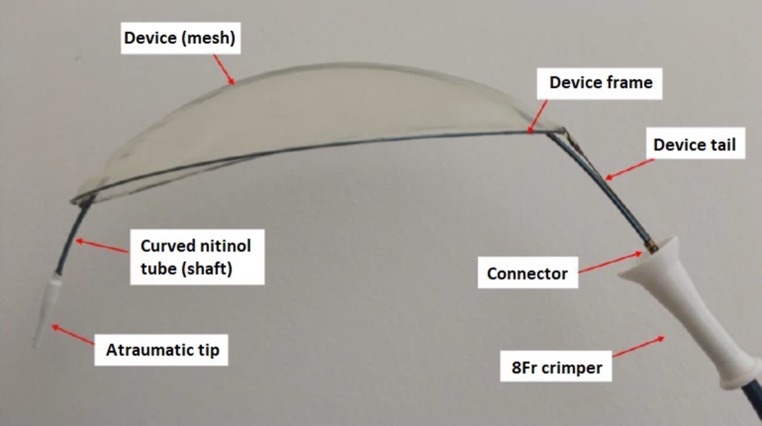
The TriGUARD 3 device

**Fig. 3:**
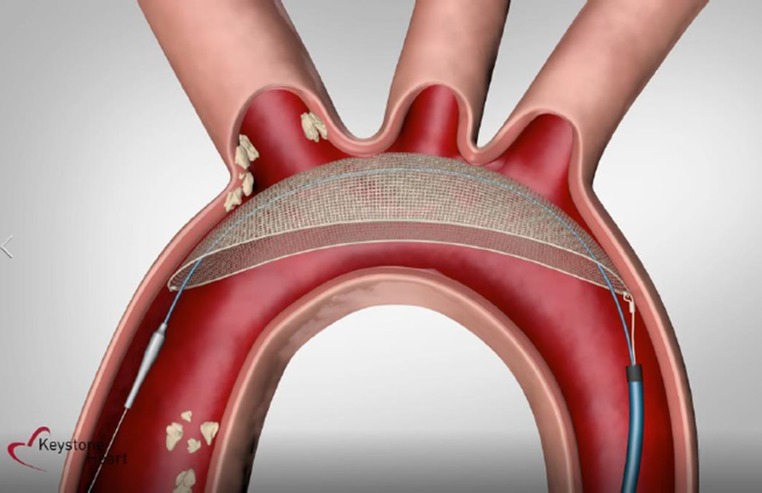
Positioning of the TriGUARD 3 device in the aortic arch

The TriGUARD 3 (Keystone Heart Ltd.) is a new-generation device that is designed to be an improvement as regards ease of use and extent of coverage over the current CE-marked TriGUARD HDH. The TriGUARD 3 shares the same basic principles of operation and has improvements such as a simplified frame design that eliminates the need for a dedicated stabiliser and that is fully visible via fluoroscopy; a reduced filter mesh pore size for deflection of smaller particles (145 × 115 µm vs. 250 × 250 µm); and a refined delivery system that reduced the delivery profile (8F instead of 9F) (Fig. 1). It is introduced transfemorally through an 8F sheath and consists of a biocompatible filter mesh on a self-stabilising frame that is positioned in the aortic arch to cover all major cerebral arteries (innominate, left carotid and left subclavian arteries). It is held in position by the circumferential pressure of the device in the aortic arch and support of the nitinol shaft (external communicating device) (Fig. 2). Once in position, debris is diverted to the descending aorta, where it is either harmless or can be treated effectively (Fig. 3). This device is not commercially available and is for investigational use only.

## Conclusion

The main expectations of a patient treated with TAVI are not only to have a reduction in symptoms and to stay alive, but also to maintain a certain lifestyle and the ability to perform daily activities. Therefore, avoiding stroke by the use of CEPD during TAVI is emerging as an important tool to reduce the burden of neurocognitive decline post-procedure. The role for such devices will further expand since TAVI has become a valid alternative in intermediate-risk patients, and it is currently under investigation in lower-risk groups. There are consistent findings in the literature supporting the use of a CEPD in terms of reducing total lesion volume, but up to now this has not translated into a reduction in hard clinical endpoints in randomised trials. Although limited in power, these findings suggest at least a favourable impact in the reduction of neurological deficits and maybe even stroke rates. From this point of view, the use of a CEPD seems beneficial at the very least. Future studies should investigate neurocognitive functioning and should even look at surrogate markers, such as HITS detected by transcranial Doppler measurements.
